# The Role of Tea Tree Oil in Alleviating Palmitic Acid-Induced Lipid Accumulation in Bovine Hepatocytes

**DOI:** 10.3389/fvets.2021.814840

**Published:** 2022-01-21

**Authors:** Tianyu Yang, Xiaoyu Ma, Maocheng Jiang, Zhiqiang Cheng, Osmond Datsomor, Guoqi Zhao, Kang Zhan

**Affiliations:** Institute of Animal Culture Collection and Application, College of Animal Science and Technology, Yangzhou University, Yangzhou, China

**Keywords:** tea tree oil, bovine hepatocytes, fatty acid metabolism, inflammation, endoplasmic reticulum stress

## Abstract

Tea tree oil (TTO) plays an important role in lipid metabolism, alleviating the inflammatory responses. Fatty liver is associated with lipid accumulation in hepatocytes, leading to inflammation. However, there is very limited information on the effects of TTO on lipid accumulation, and inflammation in bovine hepatocytes. This study aimed to evaluate whether TTO alleviates palmitic acid (PA)-induced lipid accumulation in bovine hepatocytes. Hepatocytes isolated from mid-lactating Holstein cows were pretreated with 100 μM PA for 72 h. Cells were either pretreated with PA alone (PA group) or with PA followed by 0.00625% TTO treatment for 12 h (PT group). Expression of fatty acid oxidant genes increased (*P* < 0.05) while fatty acid synthesis genes decreased (*P* < 0.05) in the PT group compared with the PA group. PA treatment resulted in increased (*P* < 0.05) expression of tumor necrosis factor-α (*TNF-*α) and interleukin-6 (*IL-6*), but these increases were less in the PT group (*P* < 0.05). Compared to the PA group, expression of phosphorylated (p)-p65 and p-inhibitor κBα (p-IκBα) was suppressed (*P* < 0.05) by TTO treatment. TTO treatment limited (*P* < 0.05) the increase in intracellular reactive oxygen species (ROS) and prevented (*P* < 0.05) a reduction in mitochondrial membrane potential observed in response to PA treatment. Expression of endoplasmic reticulum (ER) stress genes was reduced (*P* < 0.05) in the PT group compared with the PA group. Our results suggest that TTO treatment attenuates the effects of PA in hepatocytes, leading to fatty acid oxidation, decreased fatty acid synthesis, suppressed inflammatory response, and reduced ER stress. Taken together, the results of this study suggest that TTO treatment may be a promising therapeutic approach to imbalanced lipid homeostasis, inflammation and ER stress in dairy cows shortly before and after calving.

## Introduction

The transition period in dairy cattle, which spans from 3 weeks before to 3 weeks after calving, is a profoundly challenging time due to increases in energy requirements associated with gravidity and lactogenesis lead that to cattle entering a state of negative energy balance (1, 2). Drackley et al. reported that energy demands increase ~3-fold in early-lactating cattle relative to non-lactating and pregnant cattle (3). However, cows exhibit a reduction in voluntary dry matter intake during the transition period. Therefore, cattle have to mobilize fat depots to release fatty acids as an energy source to compensate for reduced energy intake.

Reynolds et al. demonstrated that excessive amounts of fatty acid are transported to the liver during the transition period (4). During lipolysis, palmitic acid (PA) participates in fatty acid release from fat depots (5). PA is a common dietary long-chain free fatty acid; it is most abundant in liver triglycerides (TGs) in both normal cows and those with steatosis. Rukkwamsuk et al. reported a significant increase in PA in dairy cows with fatty livers induced by high energy diets after parturition (5). Therefore, PA plays an important role in inducing fat deposits in the liver. When fatty acid uptake exceeds the rates of oxidation, they are stored as TGs (6). Bobe et al. reported that accumulation of high levels of fat in the liver can lead to metabolic disorders such as ketosis and fatty liver (7). Fatty liver is the most common form of chronic liver disease in high-yield dairy cows during the transition period (8–10). More than 40% of high-yield dairy cows suffer mild or moderate fatty liver and 5–10% suffer from severe fatty liver (7). Fatty liver is associated with lipid accumulation in hepatocytes, leading to endoplasmic reticulum (ER) stress, inflammation, and oxidative stress (11, 12), which cause huge economic losses (7). Therefore, understanding the molecular mechanism of fatty liver and identification of effective therapeutic strategies is of the utmost importance.

Dairy cows with fatty liver have over-active nuclear factor κB (NF-κB) in the liver, leading to elevated cytokine secretion (13). The NF-κB signal transduction pathway induces expression of genes related to pro-inflammatory cytokines via phosphorylation of inhibitor of NF-κB (IκB) induced by inhibitor κBα and IκB kinase in the cytosol (14). In addition, fatty liver in high-yielding dairy cows is associated with significantly increased inflammation and related cytokines, including interleukin-1β (IL-1β), tumor necrosis factor α (TNF-α), and interleukin-6 (IL-6) (15).

Many essential oils extracted from plants contain secondary metabolites that exhibit anti-inflammatory activity (16). Tea tree oil (TTO), an essential oil derived from the Australian plant Melaleuca alternifolia, contains more than one hundred different compounds, mainly monoterpenes and their derivatives. The main components of TTO include terpinene-4-ol, γ-terpinene, α-terpinene, 1,8-cineole, and α-terpineol (17). Previous studies have focused on the antioxidant capacity and anti-inflammatory properties of TTO (16). In addition, fragrant herbal essential oils exert a significant effect on lipid metabolism by decreasing expression of SREBP1c and associated genes involved in fatty acid synthesis (18). However, to our knowledge, there is very limited information on the effects of TTO on lipid accumulation, inflammation, and ER stress in bovine hepatocytes, and we hypothesized that TTO can limit these effects.

Therefore, the objectives of this study were to evaluate the effects of TTO on lipid metabolism, inflammation, and ER stress in bovine hepatocytes in which lipid accumulation is experimentally induced by PA treatment.

## Materials and Methods

All trials plan and methods have authorized with the Animal Ethics Committee of Yangzhou University, China. The study conducted under the Care and Use of Laboratory Animals guidelines.

### Preparation of TTO

Tea tree essential oil preliminary product were obtained from True Blue Organics (New Zealand). The TTO extract was carried out by Wuxi Chenfang Biotechnology Co., Ltd. (Wuxi, Jiangsu, China). The oil preliminary product was loaded into reaction still. Then, the nitrogen was added into the reaction still. In addition, reaction still continuously maintains the 0.2–1kg/cm2 pressure at 30–45°C, 45–70°C, and 70–85°C for 1 h, respectively. Oil pump was operated to keep the tea tree essential oils circulating in reaction still. Eventually, the cooling water was used to cool the tubes room temperature. The air compressor and nitrogen turn off, and stopping the essential oils circulating. The TTO extracts were analyzed by 9,790 gas chromatograph. The TTO major component are refer to previous paper (19). 0.1% TTO: 10 μL TTO was dissolved in 10 mL DMEM containing 0.1% dimethyl sulfoxide (DMSO). Subsequent experiments were performed by multiple dilution.

### Cell Culture

Bovine hepatocytes of three mid-lactating Holstein cows were obtained from the Institute of Animal Culture Collection and Application, Yangzhou University. Hepatocytes were isolated from each cow separately, and all assays were done with cow considered as random. The caudate liver lobe of Holstein calves was obtained through surgical liver excision according to the methods of a previous study (20). The bovine hepatocytes were digested by collagenase IV (Invitrogen, Shanghai, China) perfusion as previously described by Liu et al. (21). Briefly, the caudate lobe of the bovine liver was obtained by surgical resection. Then the liver was soaked three times in 20 mL solution A [140 mM NaCl, 10 mM N-2-hydroxyethylpiperazine-N-2-ethane sulfonic acid (HEPES), 6.7 mM KCl, 0.5 mM ethylene diamine tetraacetic acid (EDTA), and 2.5 mM glucose, pH 7.2, 37°C] and centrifuged at 120 rpm for 10 min, followed by immersion in solution B [140 mM NaCl, 30 mM (HEPES), 6.7 mM KCl, 5 mM CaCl_2_, and 2.5 mM glucose, pH 7.2, 37°C] and centrifugation at 120 rpm for 10 min. The liquid became cloudy, and the liver was perfused with collagenase IV solution (0.1 g collagenase IV dissolved in 0.5 L of perfusion solution B, pH 7.2, at 37°C) to digest the tissue. Digestion was terminated by addition of 50 mL fetal bovine serum (FBS; Gemini, Shanghai, China). The liver capsule, blood vessels, fat, and connective tissues were carefully removed using scissors and forceps. The remainder was cut into pieces and filtered successively through a 150 μm mesh and a 75 μm mesh. Hepatocytes were then washed twice with PBS at 4°C, once with red blood cell lysis buffer, and suspended in Dulbecco's modified eagle medium (DMEM) medium supplemented with 10% FBS, 100 nM insulin and 100 nM dexamethasone.

### PA/BSA Complex Solution Preparation

The PA/BSA complex solution according to a previous study (22). A 100 mM PA (Sigma-Aldrich Co, Japan) stock solution was prepared in 0.1 M NaOH by heating at 70°C in a shaking water bath. In an adjacent water bath at 55°C, a 10% (wt/vol) fatty acid-free BSA solution was prepared in DMEM medium. These two solutions were then mixed in varying proportions. For example, a 5 mM PA/10% BSA stock solution was prepared by adding 50 ml of the 100 mM PA solution dropwise to 950 ml 10% BSA solution at 55°C in a shaking water bath, then vortexing for 10 s followed by a further 10 min incubation at 55°C. The PA/BSA-complex was cooled to room temperature and passed through a sterile membrane filter (0.45 mm pore size) and stored at −20°C, where it was stable for 3–4 weeks. BSA stock solution (5 mM PA/10% BSA) was heated for 15 min at 55°C and then cooled to room temperature before use.

### Screening of PA Concentration

Hepatocytes were plated in 6-well plates at a density of 2 × 10^5^/well, and then cultured in a complete DMEM. After incubation for 12 h, hepatocytes were maintained in DMEM containing 3% BSA and treated with 0, 50, 100, or 200 μM PA for 48, 72, or 96 h, replacing the medium every 24 h. After incubation, the levels of very low-density lipoprotein (VLDL) in hepatocytes were determined using ELISA kits (Shanghai Bluegene Biotech Co. Ltd, Shanghai, China) (21), the content of ATP in hepatocytes was determined using assay kit (Nanjing Jiangcheng Biotechnology Institut, Nanjing., China), and intracellular triglycerides were assayed using a triglyceride assay kit (Applygen Technologies Inc, Beijing, China), according to the manufacturer's protocol. All the samples were assayed in triplicate.

### Screening of TTO Concentration

The cytotoxic effects of TTO on bovine hepatocytes were determined using the Cell Counting Kit-8 (CCK-8; Dojindo, Shanghai, China), according to the manufacturer's protocol. Bovine hepatocytes (5 × 10^3^ cells/well) were seeded into 96-well plates. After 12 h, bovine hepatocytes were incubated with 0.1% DMSO and either 0 (as a control), 0.001562, 0.003125, 0.006250, 0.012500, 0.025000, 0.050000, or 0.100000% TTO for 12 h. Subsequently, 10 μL CCK-8 reagent was added to cells, followed by incubation at 37°C, 5% CO_2_ for 2 h. Absorbance at 450 nm was then measured in each well-using an auto-microplate reader (Thermo scientific, Shanghai, China). All the samples were assayed in sextuplicate.

After incubation for 12 h, cells in 6-well-plates were twice rinsed gently with PBS, and then intracellular triglycerides were assayed using a triglyceride assay kit (Applygen Technologies Inc, Beijing, China). All the samples were assayed in triplicate.

### Experimental Design

**Figure 1:**
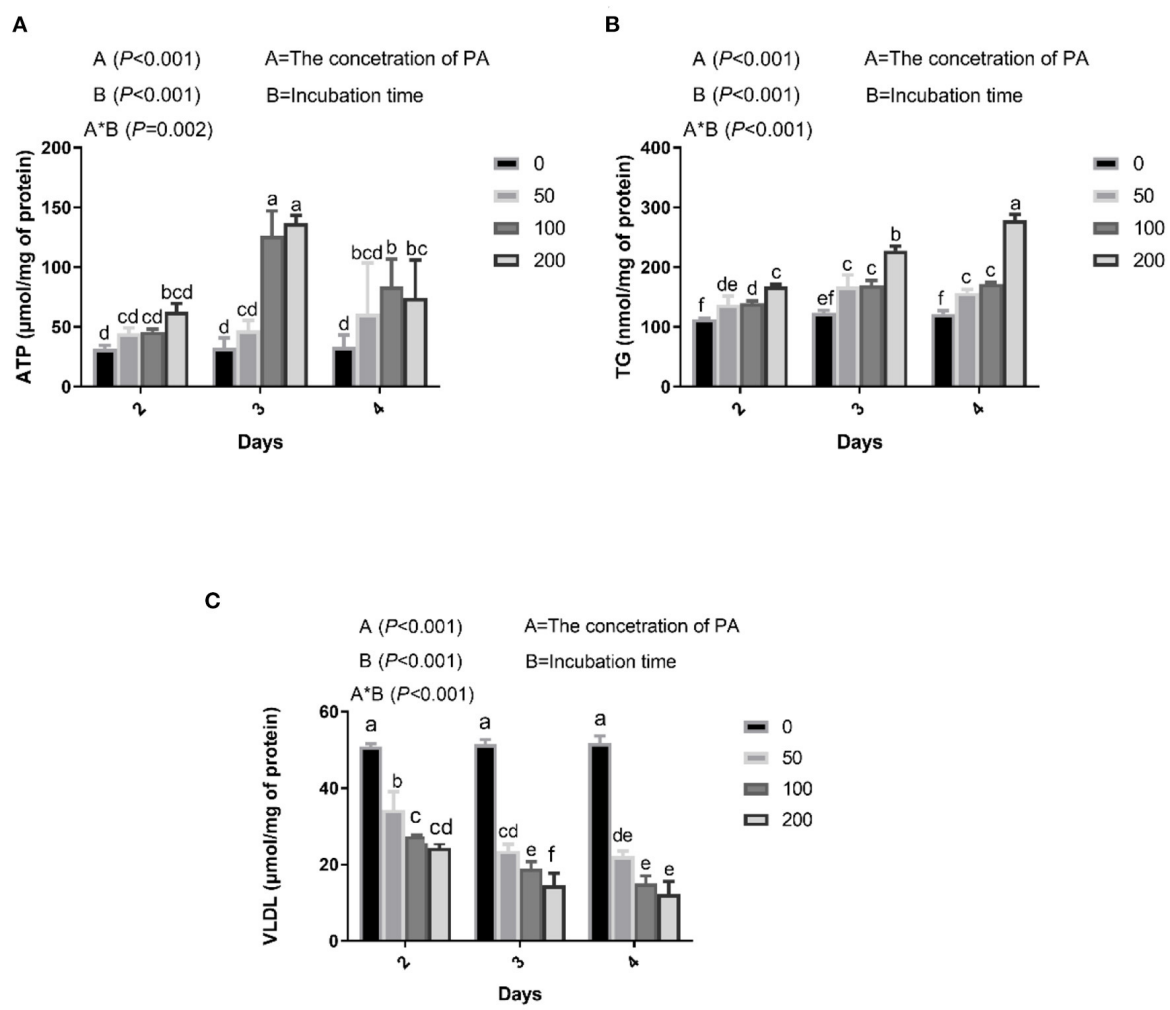
Effects of palmitic acid (PA) on mitochondria and lipid metabolism in bovine hepatocytes. Bovine hepatocytes were treated with different concentrations of PA (0, 50, 100, and 200 μM) for 48, 72, or 96 h. **(A)** Adenosine triphosphate (ATP) content in bovine hepatocytes. **(B)** Triglyceride (TG) content in bovine hepatocytes. **(C)** Very low-density lipoproteins (VLDL) content in bovine hepatocytes. Data are presented as mean ± SEM (*n* = 3). Means at the different concentration of PA indicated by different letters (a–f) differ significantly. The letters in superscript indicate that the difference between groups was significant (*P* < 0.05).

**Figure 2:**
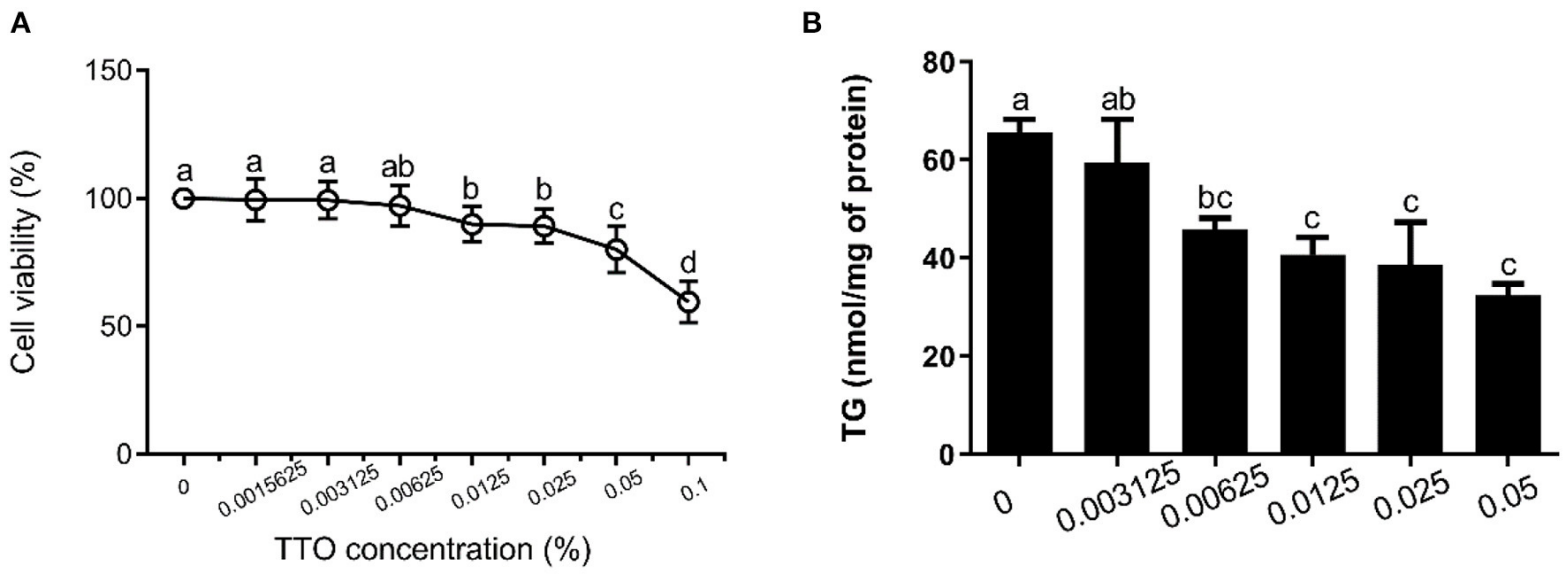
Effect of tea tree oil (TTO) on cytotoxicity and triglyceride (TG) content in bovine hepatocytes. Cells were treated with various concentrations of TTO for 12 h. **(A)** Cytotoxic effects of TTO. **(B)** The effect of TTO on TG content.

Bovine hepatocytes were seeded into 6-well plates (2.5 × 10^5^ cells/well). After 12 h, the supernatant was removed and the cells were washed three times with PBS. The hepatocytes were divided into two groups that were pretreated with different solutions for 3 days, either 3% BSA or 100 μM PA in DMEM, replacing the medium every 24 h. After 3 days of culture, discard the original culture medium. The BSA and PA groups were then each divided into two groups and treated with different solutions for 12 h, either DMEM alone (“control” and “PA” groups for the BSA and PA pretreatments, respectively) or DMEM containing 0.00625% TTO (“TTO” and “PT” groups, respectively). All the samples were assayed in duplicate. Cells were exposed to optimized doses of 100 μM PA and 0.00625% TTO according to the results of a dose-dependence assay (Figures 1, 2) in this study.

### Determination of TG Content

The cell medium was removed using a micropipettor. Hepatocytes were harvested using a cell scraper and transferred into a centrifuge tube. Then, the cells were washed twice with ice-cold PBS. The cells were lysed using lysis buffer (Applygen Technologies Inc, Beijing, China) in room temperature for 20 min. Subsequently, part of the lysate was reserved for protein quantification with BCA, and then remaining lysate was heated at 70°C for 10 min. After heating, the lysate was centrifuged for 5 min at 2,000 × g at 4°C, and the supernatant was used to determine the content of triglycerides with a commercial kit (Applygen Technologies Inc, Beijing, China), according to the manufacturer's protocol. Absorbance at 550 nm was then measured in each well-using an auto-microplate reader (Thermo scientific, Shanghai, China).

### Quantitative RT-PCR

After incubation, total RNA was isolated from the cultured cells using a TRIzol kit (Tiangen, Beijing, China). Then, the OD-1,000 + Micro-Spectrophotometer was used to measure RNA purity and concentration, with RNA quality measured via electrophoresis (2% agarose gels). In our study, the optical density (OD) 260/OD280 ratio of the total RNA was determined to be 1.9, and the intensity of the 28S ribosomal RNA band was approximately twice the intensity of the 18S ribosomal RNA band in total RNA samples, indicating that total RNA was of high quality. Reverse transcription (RT) was performed using an RT Kit (Takara, Beijing, China). RT reaction mixtures contained 1 μg total RNA and 1 × PrimeScript RT Master Mix in a final volume of 20 μL, and reactions were performed for 15 min at 37°C. Reverse transcriptase was inactivated by heating to 85°C for 5 s. qRT-PCR assays were performed using SYBR® Premix Ex TaqTM II Kit (Takara). The qRT-PCR reaction mixture contained 1 × SYBR® Premix Ex TaqTM II, 0.4 μM each forward and reverse primers, and 100 ng cDNA templates in a final volume of 20 μL, and reactions were performed as follows: initial denaturation at 95°C for 30 s, followed by 40 cycles at 95°C for 5 s and 60°C for 30 s. Before the qRT-PCR for samples, the amplification efficiencies of all primers were determined by using standard dilution series. The primers used are listed in Table 1. RefFinder (http://www.leonxie.com/referencegene.php), including Normfinder, geNorm, and the comparative ΔCT method, was used to select the first-rank reference gene (ACTB, and GAPDH) by determining the candidate genes' ranking. The final ranking was calculated by assigning an appropriate weight value to each gene, and the geometric mean of their weight values for the overall final ranking was confirmed. A higher expression stability was indicated by a lower gene geomean of ranking value. Eventually, the most stable reference gene was screened for subsequent study. In addition, GAPDH, already known to be suitable for hepatocytes (28). Therefore, GAPDH was used to normalize target gene abundance, followed by calculation of abundance using the 2^−Δ*ΔCT*^ method (29). All the samples were assayed in triplicate.

**Table 1:** Primers for real-time PCR analyses.

**Gene**	**Primer sequence, 5^**′**^−3^**′**^**	**Accession number**	**References**
SREBP1c	F: 5 GACACCACCAGCATCAACCACG 3	NM_001113302.1	(23)
	R: 5 CAGCCCATTCATCAGCCAGACC 3		
FAS	F: 5 ACAGCCTCTTCCTGTTTGACG 3	NM_001012669.1	(23)
	R: 5 CTCTGCACGATCAGCTCGAC 3		
ACC1	F: 5 TCCTGCTGCTATTGCTACTCCA 3	NM_174224.2	(23)
	R: 5 CAGTCCCCGCACTCACATAA 3		
PPARα	F: 5 TCAGATGGCTCCGTTATT 3	NM_001034036.1	(23)
	R: 5 CCCGCAGATCCTACACT 3		
CPT1A	F: 5 ACGCCGTGAAGTATAACCCT 3	NM_001304989.2	(23)
	R: 5 CCAAAAATCGCTTGTCCCTT 3		
CPT2	F: 5 CACCATTAGAAGATACCTCAGTGC 3	NM_001045889.2	(23)
	R: 5 TCCAGTTTCAAAACTCTTACACAACT 3		
IL-6	F: 5 CACCCCAGGCAGACTACTTC 3	NM_173923.2	(19)
	R: 5 TCCTTGCTGCTTTCACACTC 3		
TNF-α	F: 5 GCCCTCTGGTTCAGACACTC 3	NM_173966.3	(19)
	R: 5 AGATGAGGTAAAGCCCGTCA 3		
GPR78	F: 5 CGACCCCTGACGAAAGACAA 3	NM_001075148.1	(24)
	R: 5 AGGTGTCAGGCGATTTTGGT 3		
ATF4	F: 5 AGATGACCTGGAAACCATGC 3	NM_001034342.2	(24)
	R: 5 AGGGGGAAGAGGTTGAAAGA 3		
ATF6	F: 5 ATATTCCTCCGCCTCCCTGT 3	XM_024989876.1	(24)
	R: 5 GTCCTTTCCACTTCGTGCCT 3		
sXBP1	F: 5 TGCTGAGTCCGCAGCAGGTG 3	XM_024989876.1	(25)
	R: 5 GCTGGCAGACTCTGGGGAAG 3		
CHOP	F: 5 TGCTGAGTCCGCAGCAGGTG 3	XM_024989876.1	(26)
	R: 5 GCTGGCAGACTCTGGGGAAG 3		
EIF2A	F: 5 TCGTCATGTTGCTGAGGTCT 3	NM_175813.2 111	(27)
	R: 5 GCACCATATCCGGGTCTCTT 3		
ASK1	F: 5 GCTATGGAAAGGCAGCCAGA 3	NM_001144081.2	(27)
	R: 5 TCTGCTGACATGGACTCTGG 3		
HSP70	F: 5 GTGCAGGAGGCGGAAAAGTA 3	NM_203322.3	(27)
	R: 5 GGAAATCACCTCCTGGCACT 3		
GAPDH	F: 5 GGGTCATCATCTCTGCACCT 3	NM_001034034	(19)
	R: 5 GGTCATAAGTCCCTCCACGA 3		

*F, forward; R, reverse; NM, mRNA RefSeq; XM, predicted mRNA RefSeq; SREBP1c, sterol regulatory element binding protein-1c; FAS, fatty acid synthase; ACC1, CoA carboxylase 1; PPARα, peroxisome proliferator-activated receptor α; CPT1A, carnitine palmitoyltransferase 1A; IL-6, interleukin-6; TNF-α, tumor necrosis factor α; GPR78, 78 kDa glucose-regulated protein; ATF4, AMP-dependent transcription factor 4; ATF6, activating transcription factor-6; sXBP1, spliced X-box binding protein 1; CHOP, C/EBP homologous protein; EIF2A, eukaryotic initiation factor 2A; ASK1, apoptosis signal-regulating kinase 1; HSP70, 70-kDa heat shock protein; GAPDH, Glyceraldehyde-3-phosphate dehydrogenase*.

### Western Blotting

The target protein abundance in the hepatocytes was quantified following a published protocol (30). After incubation, the 3 × 10^6^ cells in 10 cm dish were twice rinsed gently with PBS followed by lysis using RIPA Lysis (Thermo Scientific, Shanghai, China) containing a 1 × protease inhibitor cocktail (Thermo Scientific) and 1 × phosphatase inhibitor cocktail (Roche, Shanghai, China). Protein concentrations were determined using a BCA kit (Beyotime, Beijing, China). Equal amounts (40 μg) of protein lysates were fractionated by SDS-PAGE and transferred to nitrocellulose membranes (PALL, Shanghai, China). The membranes were blocked with 5% horse serum and then incubated with primary antibody plus 5% horse serum in Tris-buffered saline with Tween (TBS-T: 10 mM Tris–HCl, pH 7.5, 150 mM NaCl, 0.05% Tween 20) using gentle shaking overnight at 4°C. Antibodies to GAPDH, phosphorylated (p)-p65, and FAS (1:1,000) were obtained from CST (Shanghai, China), to SREBP1c (1:1,000) from Novus Biologicals (USA), and p-IκBα (1:1,000) from Affinity (USA). The horseradish peroxidase (HRP)-conjugated secondary antibody was goat anti-rabbit IgG (1:5,000; CST). The target bands were detected using the Super Signal West Femto Maximum Sensitivity Substrate or Pierce ECL Plus Western Blotting Substrate (Thermo Scientific). Densitometric analysis of the bands was performed using ImageJ software (Wayne Rasband). All the densitometric values were normalized to the GAPDH (28).

### ROS Generation

2′,7′-Dichlorodihydrofluorescein diacetate (DCFH-DA) can diffuse into cells, where it is hydrolyzed to DCFH by intracellular esterase. Intracellular ROS oxidizes non-fluorescent DCFH into fluorescent DCF. Intracellular ROS in bovine hepatocytes were measured by a Reactive Oxygen Species Assay Kit containing a DCFH-DA probe (Solarbio, China), according to the manufacturer's protocol (31). Cells (4 × 10^5^ cells) were harvested and incubated with DCFH-DA at 25°C for 20 min. Fluorescence intensity was analyzed by flow cytometry (FACS LSRFortessa, USA).

### Mitochondrial Membrane Potential (ΔΨm) Analysis

The bovine hepatocytes mitochondrial membrane potential was detected by a mitochondrial membrane potential assay kit with JC-1 (Solarbio, China) following the manufacturer's protocol (31). At the end of the treatment period, cells (4 × 10^5^ cells) were collected in a polystyrene centrifuge tube and washed twice with wash buffer. Then, bovine hepatocytes were resuspended and incubated in a freshly prepared JC-1 working solution at 37°C for 20 min. Cells were analyzed using flow cytometry (FACS LSRFortessa, USA).

### Statistics

The screening of PA concentration was analyzed using two-way analysis of variance and the others results were evaluated by one-way analysis of variance (ANOVA), followed by determination of the least significant difference (LSD) for *post-hoc* multiple comparisons of treatment means, using SPSS 19.0 software (SPSS Inc.; Chicago, IL, USA). *P* values < 0.05 were considered significant.

## Results

### Lipid Accumulation in Bovine Hepatocytes After PA Treatment

We found that PA treatment gradually increased intracellular ATP, which was significantly higher in cells incubated with 100 and 200 μM PA for 3 days (Figure 1A). Culture medium containing 50, 100, and 200 μM PA significantly increased intracellular TG content after 2 days (Figure 1B) and VLDL content after 2 days (Figure 1C).

### Assessment of Cytotoxicity and Changes in TG Content in Response to TTO

The effects of TTO on cytotoxicity and TG content in bovine hepatocytes are shown in Figure 2. TTO at concentration of 0.001562, 0.003125 and 0.006250% had no cytotoxic effects on bovine hepatocytes (*P* > 0.05) (Figure 2A), and 0.006250, 0.012500, 0.025000, and 0.050000% TTO elicited a decrease (*P* < 0.05) in TG content (Figure 2B). Considering these results, 0.006250% TTO was selected for subsequent experiments.

### Effect of TTO on TG Content Following PA Treatment of Bovine Hepatocytes

**Figure 3:**
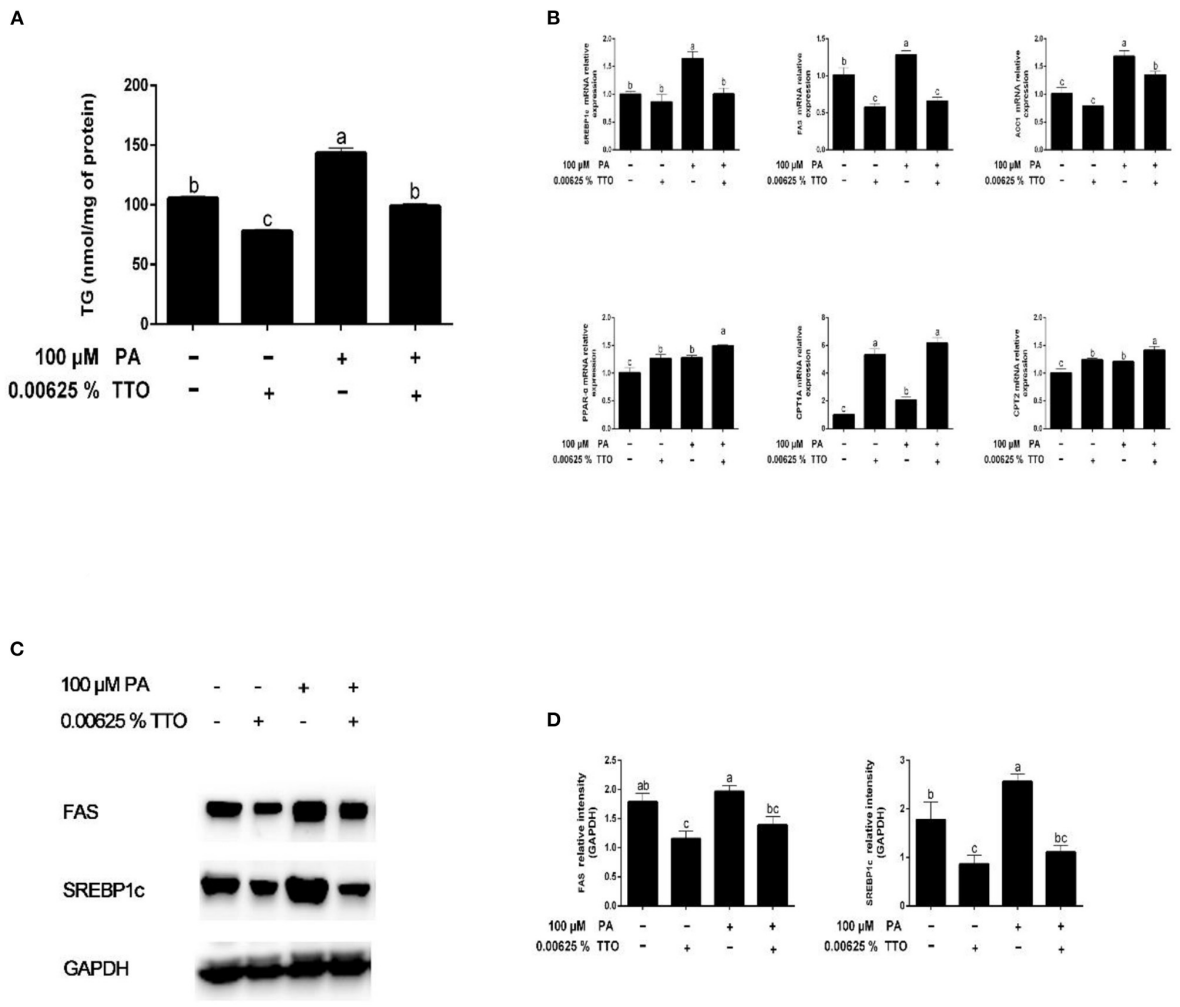
Effect of TTO on genes and proteins related to fatty acid metabolism and TG content in PA-stimulated hepatocytes. **(A)** TG content. **(B)** Expression of genes related to fatty acid metabolism, normalized to GAPDH content. **(C,D)** Intensity of bands in immunoblots **(C)** and images of blots **(D)**. Protein expression was normalized to GAPDH content. All results are expressed as mean ± SEM. These data are representative of three independent experiments.

The changes in TG concentration in bovine hepatocytes treated with PA are shown in Figure 3A. Compared with the control group, TG concentration significantly decreased (*P* < 0.05) in response to TTO incubation, while it increased in response to PA. TTO treatment elicited a reduction (*P* < 0.05) in TG content in bovine hepatocytes pretreated with PA.

### Effect of TTO on the Expression of Genes Related to Fatty Acid Metabolism, Inflammation, and ER Stress in Bovine Hepatocytes Treated With PA

Compared with the control group, PA stimulation increased (*P* < 0.05) expression of synthesis-related genes, including *SREBP1c, FAS*, and *ACC1*, and fatty acid oxidation genes (*PPAR-*α, *CPT1A*, and *CPT2*) (Figure 3B). TTO treatment resulted in downregulation of *FAS* gene expression (*P* < 0.05) and upregulation of *PPAR-*α and *CPT1A* gene expression (*P* < 0.05) in bovine hepatocytes compared with the control group. Moreover, PT treatment exhibited decreased (*P* < 0.05) expression of *FAS* and SREBP1c genes and increased (*P* < 0.05) expression of *PPAR-*α and *CPT1A* genes.

**Figure 4:**
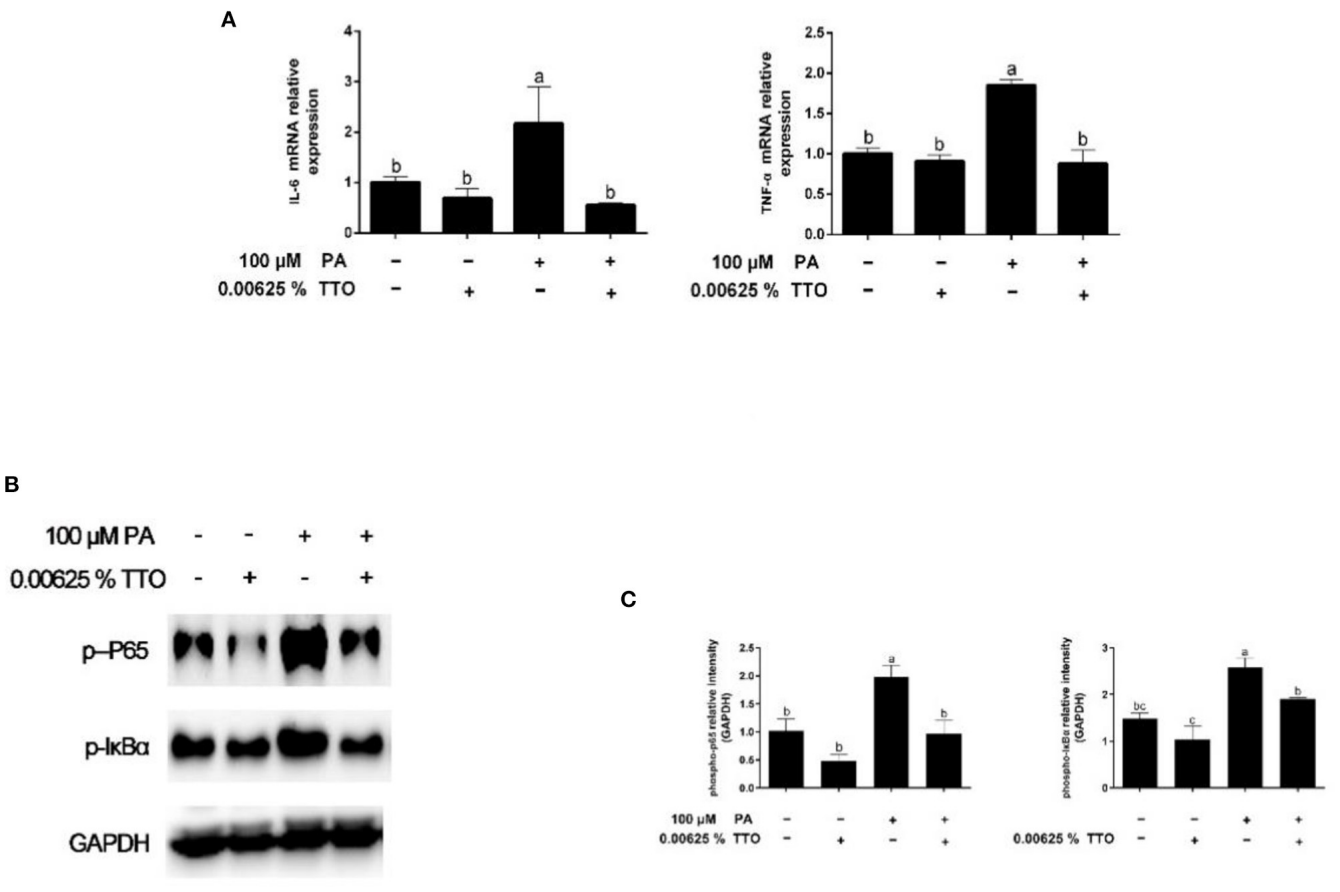
Expression of genes and proteins related to inflammation in bovine hepatocytes treated with TTO (0.00625%) and/or PA (100 μM). **(A)** Expression of genes related to inflammatory response, normalized by GAPDH. **(B,C)** Immunoblots and corresponding intensities of the bands. Protein expression was normalized by the respective abundance of GAPDH. All results are expressed as the mean ± SEM. The letters above the error bars indicate that the differences between groups was significant (*P* < 0.05). These data are representative of three independent experiments.

Expression of genes related to the inflammatory response is shown in Figure 4A. PA stimulation elicited an increase (*P* < 0.05) in *IL-6* and *TNF-*α gene expression compared with the control group. PT group triggered (*P* < 0.05) expression of *IL-6* and *TNF-*α genes in hepatocytes compared to that in PA-treated cells.

**Figure 5:**
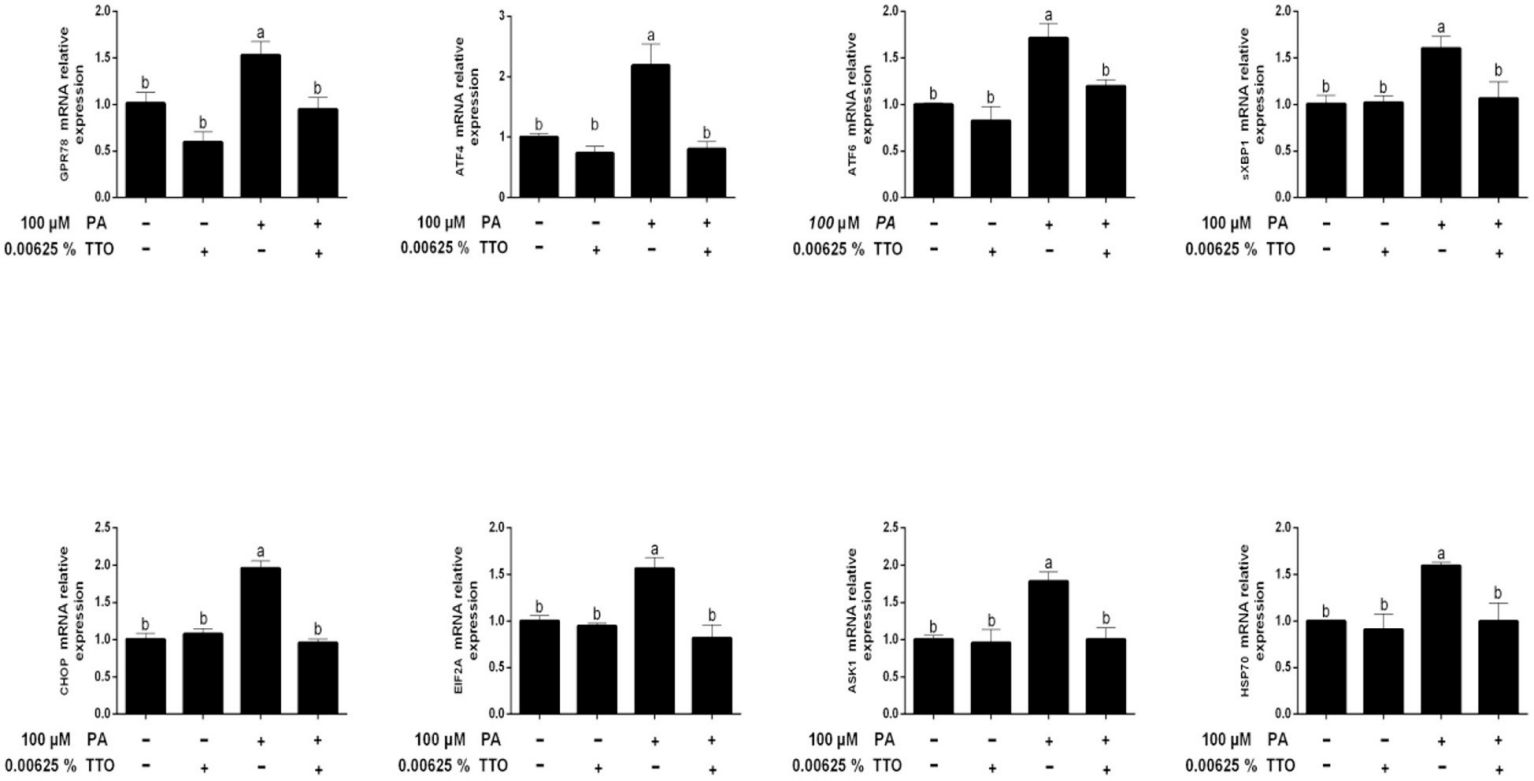
Expression of genes related to endoplasmic reticulum stress in bovine hepatocytes treated with TTO (0.00625%) and/or PA (100 μM). Expression of genes related to endoplasmic reticulum stress, normalized by GAPDH. All results are expressed as the mean ± SEM. The letters above the error bars indicate that the difference between groups was significant (*P* < 0.05). These data are representative of three independent experiments.

Figure 5 shows that compared with the control group, PA stimulation specifically triggered (*P* < 0.05) gene expression of the 78 kDa glucose-regulated protein (*GPR78*), AMP-dependent transcription factor 4 (*ATF4*), activating transcription factor-6 (*ATF6*), spliced X-box binding protein 1 (*sXBP1*), C/EBP homologous protein (*CHOP*), eukaryotic initiation factor 2A (*EIF2A*), apoptosis signal-regulating kinase 1 (*ASK1*), and 70-kDa heat shock protein (*HSP70*). In addition, compared with control group (*P* > 0.05), TTO had no effect on the expression of genes related to ER stress. PT treatment exhibited decreased (*P* < 0.05) gene expression of *GPR78, ATF4, ATF6, sXBP1, CHOP, EIF2A, ASK1*, and *HSP70*, compared with that in PA-stimulated hepatocytes.

### TTO Regulated Expression of Proteins Related to Fatty Acid Metabolism in PA-Stimulated Bovine Hepatocytes

We assessed protein expression of FAS and SREBP1c by using western blotting (Figures 3C,D). Expression of proteins related to fatty acid synthesis, including FAS and SREBP1c, was reduced (*P* < 0.05) in the TTO group compared with the control group, but increased in the PA group. PT group exhibited decreased (*P* < 0.05) protein expression of FAS and SREBP1c compared with the PA-stimulated group.

### PA-Stimulated Expression of Proteins Associated With NF-κB Signaling Was Inhibited by TTO Treatment

Levels of phosphorylated IκBα and phosphorylated p65 are shown in Figures 4B,C. The levels of p-IκBα and p-p65 in hepatocytes increased in response to PA stimulation, while TTO reduced (*P* < 0.05) protein expression of p-IκBα and p-p65 compared to that in the control group. Compared with the PA-treatment group, PT treatment reduced (*P* < 0.05) expression of the phosphorylated forms of IκBα and NF-κB.

### TTO Stabilized Intracellular ROS Levels and Mitochondrial Membrane Potential in Bovine Hepatocytes Stimulated With PA

**Figure 6:**
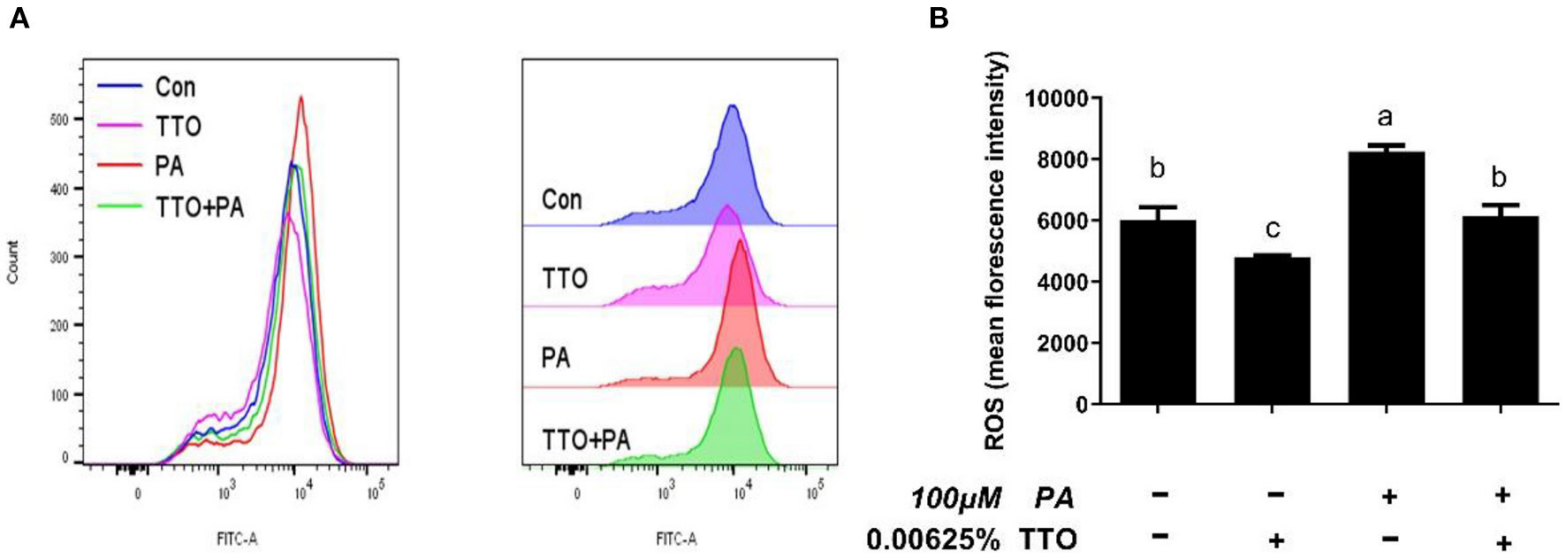
The effect of TTO on ROS production. **(A)** ROS levels in bovine hepatocytes treated with TTO (0.00625%) and/or PA (100 μM). Cellular ROS generation following different treatments was measured by staining with DCFH-DA. The fluorescence was detected using flow cytometry. **(B)** Cellular ROS level. All results are expressed as the mean ± SEM. The letters above the error bars indicate that the difference between groups was significant (*P* < 0.05). These data are representative of three independent experiments.

**Figure 7:**
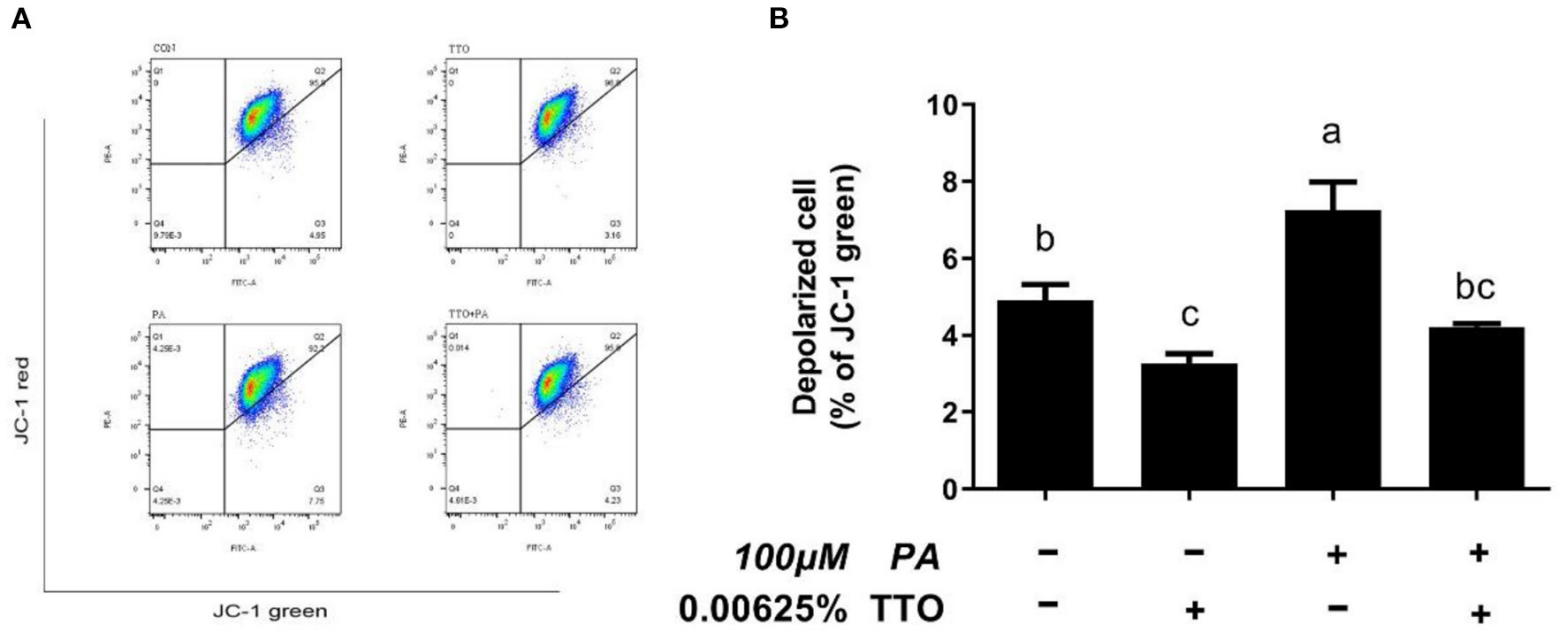
The effect of TTO on mitochondrial membrane potential. **(A)** Mitochondrial membrane potential in bovine hepatocytes treated with TTO (0.00625%) and/or PA (100 μM). Mitochondrial membrane potential following the different treatments was measured by staining with JC-1. The fluorescence was detected using flow cytometry. The number of depolarized cells is indicated as a percentage of the total cells. **(B)** The mitochondrial membrane potential percentages in cultures exposed to TTO or PA. All results are expressed as the mean ± SEM. The letters above the error bars indicate that the difference between groups was significant (*P* < 0.05). These data are representative of three independent experiments.

To define antioxidant levels in an inflammatory environment and determine the functional status of mitochondria under oxidative stress, we measured intracellular ROS levels and mitochondrial membrane potentials in bovine hepatocytes (Figures 6A,B, 7A,B). Treatment with TTO reduced (*P* < 0.05) intracellular ROS levels, while PA stimulation increased (*P* < 0.05) ROS levels compared with control group. In addition, PT group exhibited reduced (*P* < 0.05) intracellular ROS levels compared with the group treated by PA alone.

In healthy cells characterized by high mitochondrial membrane potential, JC-1 forms aggregates within mitochondria and emits red fluorescence. However, when cells have a low mitochondrial membrane potential, JC-1 remains monomeric in the cytoplasm and emits green fluorescence (32). Stimulation with PA lead to a collapse in mitochondrial membrane potential in bovine hepatocytes, but PT group prevented this change (*P* < 0.05).

## Discussion

During the transition period, dairy cattle experience tremendous physiological, metabolic, immunological, and nutritional changes (33, 34). To meet their energy demands, lipid mobilization of adipose tissue is an efficient physiological adaptation in dairy cattle (35). Increased lipid mobilization leads to an increase in blood fatty acids, which are absorbed by the liver, leading to TG accumulation in liver and an enhanced inflammatory response accompanied by ER stress (34, 36).

High levels of circulating saturated fatty acids are linked with fatty liver, obesity, and hyperlipidemia. The major circulating fatty acid is saturated PA (C16:0), which has been implicated in lipid accumulation (37). In addition, PA is a 16-carbon saturated fatty acid that is the first produced during lipogenesis. In this study, we chose to utilize PA treatment to induce lipid accumulation in bovine hepatocytes as a model of fatty liver. ATP production and TG concentration is higher, and VLDL is lower, in fatty liver compared with that in healthy cows (21, 37). Our study showed that after incubating bovine hepatocytes with 100 μM PA for 3 days, there were significant increases in intracellular levels of TG and ATP, and a decrease in intracellular VLDL. Based on these results, bovine hepatocytes were stimulated with 100 μM PA for 3 days in subsequent experiments.

Fragrant herbal essential oils can be beneficial for lipid metabolism (18). Our previous study demonstrated that TTO contains terpinen-4-ol, α-terpinene, α-terpineol, α-pinene, p-cymene, and 1,8-cineole, γ-terpinene, terpinolene, and pinocarveol (19). The phytochemical content of p-cymene, which is harmful to humans and animals, is <0.05%. Notably, the content of terpinen-4-ol, which can inhibit secretion of cytokines such as IL-1β, TNF-α, and IL-10, is ~60.23 % (38). In addition, previous studies showed that TTO can play an important role as an anti-inflammatory agent (16). The present results showed that <0.00625% TTO did not impair cell viability of bovine hepatocytes and more than 0.00625% TTO elicited a remarkable decrease in TG content. Notably, 0.00625% TTO can also reduce TG concentration in PA-stimulated bovine hepatocytes. Therefore, TTO is a potential treatment option for reducing lipid accumulation induced by PA in bovine hepatocytes.

Fatty liver can occur when hepatic lipid synthesis exceeds the rate of oxidation and secretion of lipids by the liver (7). Compared with absorption of fatty acids, their synthesis in the liver has less effect on production of milk fat in the mammary glands of bovines. Fatty liver may be caused by a disturbance of the energy balance of dairy herds. Therefore, the regulation of liver lipid homeostasis plays an important role in maintaining normal physiological function. Interestingly, addition of TTO to PA-stimulated hepatocytes reduced cellular TG content to the same level as in the control group. Du et al. reported that hepatic lipid synthesis and oxidation were triggered in dairy cows with mild fatty liver (37). SREBPs are transcription factors that promote expression of genes related to lipogenesis and fatty acid synthesis (39). SREBP1c is one member of this family that may modulate many genes related to lipid synthesis and deposition (40), including *ACC1* and *FAS*, which are necessary for fatty acid synthesis in the liver, white fat, and other tissues (41). The incidence of fatty liver increases when expression of SREBP1c and its target genes, *FAS* and *ACC1*, is significantly increased in bovine hepatocytes (42). In addition, PPARα plays a vital role in regulation of mitochondrial and fatty acid oxidation in ruminants, including regulation of downstream targets such as *CPT1A* and *CPT2* (43). In this study, we demonstrated that hepatic synthesis and oxidation of lipids increased in bovine hepatocytes stimulated by PA, which is consistent with a previous study (37). Fragrant herbal essential oils reduce TG content by inhibiting expression of SREBP1c and its target genes involved in fatty acid synthesis (18). As expected, we found that the gene and protein of SREBP1c were remarkably reduced in the PT group. Interestingly, expression indexes of the downstream genes, including *FAS* and *AAC1*, were consistent with a change in SREBP1c expression. However, gene expression of PPARα and its downstream protein targets, *CPT1A* and *CPT2*, increased. Taken together, these results suggest that addition of TTO promotes lipid oxidation and inhibits lipid synthesis by regulating expression of key enzymes in bovine hepatocytes stimulated by PA.

Accumulation of TGs is strongly associated with inflammation, ER stress, and oxidative stress (1, 12). Our results show that accumulation of TG over time can activate the NF-κB pathway and aggravate inflammation, which is consistent with a previous study (44). It has been demonstrated that TTO exhibits anti-inflammatory and anti-oxidative activity properties (16, 23). NF-κB plays a vital role in intracellular regulation of inflammation and the immune response (45, 46). As a cytoplasmic part of the NF-κB complex, IκBα initiates NF-κB activity primarily by inhibiting nuclear localization of the NF-κB complex and inducing phosphorylation of NF-κB p65 (47). NF-κB is a widespread transcription factor implicated in the activation of many genes, including those involved in alcoholic liver injury (48). In our previous study, TTO inhibited NF-κB in goat rumen epithelial cell (49). Our immunoblotting results show that adding TTO prevents NF-κB and IκB phosphorylation. NF-κB activity is linked with liver inflammation (50). Following activation, NF-κB causes expression of inflammatory cytokines, including TNF-α and IL-6 (48). Our result show that TTO greatly decreased expression of IL-6 and TNF-α genes in PA-stimulated bovine hepatocytes. Activation of NF-κB can directly or indirectly cause cellular damage by inducing oxidative stress, DNA damage, and lipid peroxidation (51), which lead to cell membrane damage, including mitochondrial fission and eventually cell death. Consequently, it is vital to reduce cellular damage caused by oxidative stress. As expected, TTO supplementation reduced ROS generation in PA-treated bovine hepatocytes, thus protecting lipid bilayers. ROS accumulation leads to collapse of the mitochondrial membrane potential, which is vital for mitochondrial function. Reduced mitochondrial membrane potential promotes mitochondrial permeability by opening pores and allowing release of cytochrome c from mitochondria into the cytosol, which activates the caspase cascade pathway that induces apoptosis (52–54). We observed that treatment with TTO prevented lipid accumulation resulting from decreased ROS production by preserving mitochondrial membrane potential. Our research suggests that treatment of bovine hepatocytes with TTO can prevent NF-κB activity, suppress pro-inflammatory cytokines and ROS generation, and preserve mitochondrial membrane potential.

Generation of ROS is associated with ER stress. In addition, high levels of fatty acid directly induce ER stress in bovine hepatocytes (55, 56), which precipitates hepatic lipogenesis (57–59). Intriguingly, high levels of fatty acids have been shown to increase lipid accumulation in calf hepatocytes (13), consistent with our research. Earlier studies focused on a causative role of ER stress in hepatocytes, and here we tested whether TTO could relieve ER stress in bovine hepatocytes. GPR78 and CHOP are markers of ER stress (60), and we observed that exposing bovine hepatocytes to PA significantly increased expression of the *GPR78* and *CHOP* genes. As predicted, treating these PA-stimulated hepatocytes with TTO reduced *GPR78* and *CHOP* expression. When unfolded proteins accumulate in the ER rumen, chaperone proteins detach from the ER membrane and bind to them. In addition, delayed separation of chaperone proteins can activate the ER membrane protein GRP78. Reaction with unfolded proteins is initiated by aggregation and activation of transmembrane proteins, leading to activation of three classical transmembrane proteins (PERK, IRE1, and ATF6) (61). The PERK-EIF-2A-ATF4, ATF6, and IRE1-XBP pathways all activate the downstream *CHOP* gene. In addition, HSP70, a molecular chaperone, can induce a response to cellular stressors (62). The present study revealed that expression of *EIF-2A* (downstream of PERK), *ATF4* (downstream of EIF-2A), *ASK1* (downstream of IRE1), *ATF6, CHOP*, and *HSP70* was triggered at the mRNA level by PA treatment of hepatocytes, and that TTO treatment reversed this effect.

In conclusion, our results show that TTO exerts anti-inflammatory effects and acts to promote lipid homeostasis in bovine hepatocytes. These effects are associated with inhibition of NF-κB signaling and SREBP1c expression. Taken together, the results of this study suggest that TTO may be a promising supplement for treatment of imbalanced lipid homeostasis, inflammation and ER stress in dairy cows during the transition period.

## Data Availability Statement

The original contributions presented in the study are included in the article/supplementary material, further inquiries can be directed to the corresponding author/s.

## Ethics Statement

This experiment was performed under protocols approved by the Animal Care and Use Committee of Yangzhou University, Yangzhou, China and Ethic code is DWLL-202011-201. The authors confirm that they have followed EU standards for the protection of animals used for scientific purposes. Written informed consent was obtained from the owners for the participation of their animals in this study.

## Author Contributions

TY performed experiment work, analyzed the data, and wrote the manuscript. XM also performed experiment work. MJ revised the manuscript. KZ and ZC: writing-reviewing and editing. KZ and GZ provide the support of funding. KZ contributes to the experimental idea. All authors contributed to the article and approved the submitted version.

## Funding

This study was supported by the National Natural Science Foundation of China (No. 32002200), the Research Project of Natural Science Foundation of Jiangsu Province (BK20190898), and China Agriculture Research System of MOF and MARA.

## Conflict of Interest

The authors declare that the research was conducted in the absence of any commercial or financial relationships that could be construed as a potential conflict of interest.

## Publisher's Note

All claims expressed in this article are solely those of the authors and do not necessarily represent those of their affiliated organizations, or those of the publisher, the editors and the reviewers. Any product that may be evaluated in this article, or claim that may be made by its manufacturer, is not guaranteed or endorsed by the publisher.
